# Compliance with spectacle wear among learners with hearing impairment in Ghana

**DOI:** 10.4102/ajod.v13i0.1314

**Published:** 2024-06-13

**Authors:** Michael A. Kwarteng, Khathutshelo P. Mashige, Samuel Kyei, Pirindhavellie Govender-Poonsamy, Daniel S.Q. Dogbe

**Affiliations:** 1 Department of Optometry, Faculty of Science and Engineering, Bindura University of Science Education, Bindura, Zimbabwe; 2 Discipline of Optometry, School of Health Science, University of KwaZulu-Natal, Durban, South Africa; 3 Optometry Unit, Department of Clinical Surgical Science, Faculty of Medical Sciences, The University of the West Indies, St. Augustine Campus, West Indies, Trinidad and Tobago; 4 Department of Optometry and Vision Science, School of Allied Health Sciences, University of Cape Coast, Cape Coast, Ghana; 5 Department of Special Education, Faculty of Education, University of Education, Winneba, Ghana

**Keywords:** spectacle compliance, hearing impairment, visual impairment, refractive error, Ghana

## Abstract

**Background:**

Hearing-impaired learners with refractive problems require correction because poor vision hinders their development and educational pursuits.

**Objectives:**

To determine the level of compliance with spectacle wear in learners with hearing impairment in Ghana.

**Method:**

A descriptive cross-sectional study design was used to investigate the level of compliance with spectacle wear in hearing-impaired learners with uncorrected refractive errors (URE). The participants were from six schools for the hearing impaired, comprising three schools from each sector (Northern and Southern) of Ghana.

**Results:**

Of the 1914 learners screened, 69 (3.61% CI: 2.82–4.54%) had URE. Sixty-two (89.9%) learners with URE had myopia (-0.50 Dioptre Sphere (DS) to -2.00DS), and 7 (10.1%) had hyperopia (+2.00DS to +10.00DS). There were more females (53.6%) with URE than males, and their ages ranged from 8 to 35 years, with a mean of 17.35 ± 5.19 years. Many (56.5%) learners complied with spectacle wear after 3 months of reassessment, with females being more compliant than males, but the difference was not significant (*p* = 0.544). Learners who complied well with the spectacle wear were those with moderate visual impairment (VI), followed by mild VI, while those with no VI were the least compliant. A significant difference was observed between spectacle compliance and presenting VI (*p* = 0.023).

**Conclusion:**

The spectacle wear compliance level was high compared to a previous study (33.7%) in Ghana.

**Contribution:**

This study highlights the importance of addressing URE among learners with hearing impairment in Ghana and Africa.

## Introduction

Refractive errors have been identified as a significant source of visual impairment (World Health Organization 2022). Learners with refractive problems require special attention because poor vision hinders their development and educational pursuits (Woodhouse et al. 2013). Uncorrected refractive error (URE) among learners has been associated with decreased self-esteem and poor reading abilities (Pirindhavellie et al. 2023). Refractive errors can be corrected with the use of contact lenses, spectacles and refractive surgeries (Kwarteng, Katsvanga & Kyei 2022a; Naidoo et al. 2016). Although spectacles are a relatively cost-effective intervention, middle- and low-income countries still battle to provide this service because of access and cost implications (Ilechie et al. 2019).

Refractive error corrections can significantly improve children’s wellbeing, quality of life and academic performance (Kwarteng et al. 2024; Pirindhavellie et al. 2023). However, eye care corrective devices such as spectacles are not always utilised even if they are available, provided and accessible (Ezinne et al. 2020; Ilechie et al. 2019). Poor compliance with spectacle wear has been reported among school children (Ilechie et al. 2019). Some of the reasons cited for non-compliance with spectacle wear among learners included the spectacles not being a good fit, disapproval from others, spectacles being prescribed for low magnitude refractive errors, disapproval of spectacles by parents, inconvenience with certain activities and constantly forgetting the spectacles at home (Du et al. 2023; Ezinne et al. 2020; Ilechie et al. 2019). Having these factors addressed will lead to improved compliance with spectacles.

Learners with hearing impairment rely primarily on visual cues for communication and learning (Kwarteng et al. 2022c; Pehere et al. 2019). A high prevalence of VI has been reported among learners with hearing impairment in sub-Saharan Africa, with URE being the leading cause (Kwarteng et al. 2022c). To achieve the goal of reducing the burden of VI because of URE, there is a need for comprehensive eye screening, the provision of spectacles and assessing spectacle utilisation among these learners. Therefore, it is important to prioritise the eye health of learners with hearing impairment and provide them with the necessary interventions to improve their visual abilities and learning outcomes.

While various methods for offering free or inexpensive spectacles are being conducted, efforts to offer these services will be ineffective if there is poor compliance with spectacle wear (Pavithra, Hamsa & Madhukumar 2014). In Ghana, only one study has investigated the provision of spectacles and determined the compliance level with spectacle wear among pupils in mainstream schools (Ilechie et al. 2019). No information could be found on the compliance with spectacle wear among learners with disabilities such as hearing impairment in Ghana and beyond. With the high prevalence of VI among learners with hearing impairment in Ghana (Kwarteng et al. 2022b, 2022c), it was imperative to consider their visual needs and provide them with solutions. This study aimed to determine the level of compliance with spectacle wear among learners with hearing impairment who were provided with spectacles for the correction of refractive errors in Ghana schools for the hearing impaired.

## Research methods and design

A descriptive cross-sectional study design was used to investigate the level of spectacle compliance among learners with hearing impairment. This study was conducted in six schools for learners with hearing impairment, three schools from the Southern part of Ghana and the other three from the Northern part of the country. Ghana is located in the West of Africa with an estimated population of about 31 million with 16 administrative regions and two sectors (northern and southern) (Ghana Statistical Service 2021). There are 11 public schools for learners with hearing impairment in Ghana with an estimated student population of 3000 (Oppong & Fobi 2019).

A stratified random sampling method was employed to randomly select six schools from the total of 11 public schools for the hearing impaired nationwide. The schools were classified into two sectors based on the national geographic classification: the northern sector, consisting of four schools, and the southern sector, consisting of seven schools. Three schools were chosen at random from a pool of four in the northern sector. The chosen schools were Gbeogo, Ashanti and Savelugu Schools for the hearing impaired. In the southern sector, schools were classified as one if their distance was less than 80 km. By applying this criterion, four schools were consolidated into a single entity, resulting in the southern schools being reduced to four. Out of the four available sites, three schools were selected at random to serve as the locations for this study. The chosen schools are Cape Coast, Sekondi and State Schools for the hearing impaired.

The study utilised a convenient purposive sampling to choose the participants, as it required the inclusion of all learners who were present during the study period and were prescribed spectacles. The study used ready-made (pop-in) spectacles for learners with myopia and custom-made spectacles for learners with hyperopia.

## Theoretical framework

**FIGURE 1:**
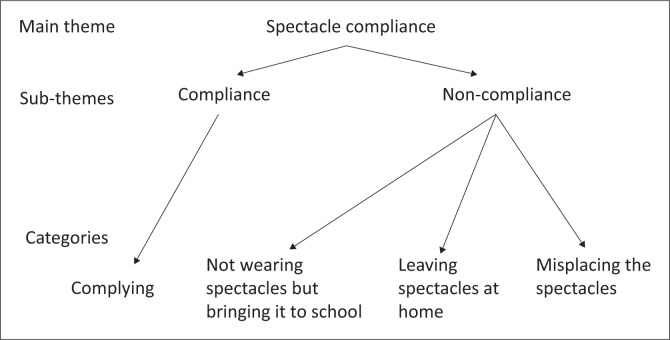
Theoretical framework for spectacle compliance.

The theoretical framework utilised in this study was adopted from Pavithra et al. (2014) study conducted among learners in stream schools. Two major themes were used in this study, with the first focusing on learners who willingly complied with wearing spectacles, while the second explored those who did not comply with wearing them. These themes allowed for a comprehensive examination of the factors influencing compliance and non-compliance behaviours about spectacle usage. Three subthemes emerged from those not complying, including not wearing them but bringing them to school, leaving the spectacles at home and misplacing them (Figure 1).

### Data collection

Data were collected with a well-designed data collection tool with different sections modified from a validated data collection tool and a last section as a questionnaire on spectacle compliance (Pavithra et al. 2014). The first section covered demographic data such as age and sex, among others while the second section measured visual acuity and examined anterior and posterior segments of the eye as described in a preceding study (Kwarteng et al. 2022b). An objective assessment of refractive status was carried out with the use of the dry retinoscopy technique (Pavithra et al. 2014), after which participants were provided spectacles with different colours and shapes to choose from. Refractive status was determined using spherical equivalents and defined as myopia ≥ –0.50 DS and hyperopia ≥ +2.00 DS (Kwarteng et al. 2022a; Tagoh et al. 2020).

Compliance with spectacle wear was determined with the assistance of teachers using a structured questionnaire with the learners from May 2022 to December 2022, while the entire study’s data collection period was from March 2022 to December 2022. Before distributing the spectacles, learners and their teachers were educated on the advantages and disadvantages of spectacle wear, such as improvement in visual functions, protection of the eyes from dirt and harmful radiations and initial wear discomfort, among others (Pavithra et al. 2014). Unscheduled visits were made to the schools 3 months after the distribution of full-time spectacles to observe compliance with spectacle wear. On the day of the assessment, learners not wearing the spectacles were questioned on the whereabouts of the spectacles and the reasons for not wearing them. The interviews were conducted on a class-to-class basis, starting with classes that were closer to the school entrance. Compliance was defined as the learner wearing the spectacles on the assessment day and confirmation from class teachers that the learner had been wearing the spectacles during school hours (Brien Holden Vision Institute 2019; Gajiwala et al. 2021; Ilechie et al. 2019).

Uncorrected visual acuity (UCVA) was defined using the World Health Organization’s definition for visual impairment, with moderate VI (less than 6/18–6/60), mild VI (less than 6/12–6/18) and no VI (6/6–6/12) (World Health Organization 2022).

### Ethical considerations

Ethical approval was obtained from the Biomedical Research Ethics Committee (BREC) of the University of KwaZulu-Natal (Reference number: BREC/00003247/2021) and the Ghana Health Service Ethical Review Committee (Reference number: GHS-ERC: 006/04/21). The study adhered to the Declaration of Helsinki’s guidelines involving the use of human subjects. Informed consent was obtained after each learner and their parents or guardians were informed about the study’s purpose. There were no risks and/or discomforts associated with taking part in the study, and the students received no financial compensation. The study was voluntary, and participants were informed that they could discontinue at any time without incurring any fees or losing access to treatment or other benefits to which they would ordinarily be entitled.

### Data analysis and management

Data from the study were analysed using the SPSS version 21 (SPSS Inc, Chicago, USA). Kernel density plots were constructed to evaluate the normality of the data. Means, standard deviations, prevalence and 95% confidence intervals were determined. A chi-square test was conducted to assess the relationship between categorical variables (*p* < 0.05). The data will be shredded 5 years after the completion of the study. The data will be exclusively accessible to the researchers because of its encryption in a password-protected computer.

## Results

A total of 1914 learners were screened during the study period, with 69 (3.61% CI: 2.82–4.54%) having uncorrected refractive error, making the 69 learners the sole participants in this study. The majority (89.9%) of the learners were classified as having myopia (–0.50DS to –2.00DS) and the rest hyperopia (+2.00DS to +10.00DS), with females (53.6%) being the predominant group. There was no statistically significant difference between the proportions of males and females (χ^2^ = 0.71, *df* = 1, *p* = 0.399). Learners’ ages ranged from 8 to 35 years, with a mean of 17.35 ± 5.19 years. Learners from the southern sector of Ghana formed the majority (62.3%), and there was a statistically significant difference between the proportions of learners from the two regions (χ^2^ = 8.29, *df* = 1, *p* = 0.004). However, there was no statistically significant difference in the proportions of sex between the two regions (Table 1). A chi-square test revealed no statistically significant association between variables (region, school and VI before correction) and sex (*p* > 0.05).

**TABLE 1:** Distribution of demographics according to sex.

Variables	Sex of learners				Total	%	*p*
	Male		Female				
	*n*	%	*n*	%			
Area							0.213
Northern	15	21.7	11	15.9	26	37.7	-
Southern	17	24.6	26	37.7	43	62.3	-
School							0.351
Jamasi	8	11.6	8	11.6	16	23.2	-
Cape Coast	6	8.7	10	14.5	16	23.2	-
Savelugu	2	2.9	1	1.4	3	4.3	-
Sekondi	6	8.7	4	5.8	10	14.5	-
Gbeogo	5	7.2	2	2.9	7	10.1	-
Tema	5	7.2	12	17.4	17	24.6	-
Uncorrected visual acuity (UCVA)							0.634
Normal (6/6–6/12)	12	17.4	10	14.5	22	31.9	-
Mild (less than 6/12–6/18)	9	13.0	13	18.8	22	31.9	-
Moderate (less than 6/18–6/60)	11	15.9	14	20.3	25	36.2	-

**Total**	**32**	**46.4**	**37**	**53.6**	**69**	**100.0**	**-**

Note: Significant at *p* < 0.05.

### Distribution of compliance with spectacles according to sex

Most (56.5%) of the learners complied with wearing spectacles, and among them, females complied more than males; however, the difference was not statistically significant (χ^2^ = 2.495, *df* = 1, *p* = 0.114). More males than females reported misplacing their spectacles. Overall, there was no significant difference between spectacle compliance and sex (χ^2^ = 2.14, *df* = 3, *p* = 0.544) as shown in Table 2.

**TABLE 2:** Distribution of compliance with spectacles according to sex.

Compliance with spectacle wear	Sex of learners				Total	%	*p*
	Male		Female				
	*n*	%	*n*	%			
Wearing spectacles	16	50	23	62.2	39	56.5	-
Not wearing but brought them to school	5	15.6	3	8.1	8	11.6	-
Left them at home	5	15.6	7	18.9	12	17.4	-
Misplaced them	6	18.8	4	10.8	10	14.5	-

**Total**	**32**	**100**	**37**	**100**	**69**	**100.0**	**0.544**

Note: Significant at *p* < 0.05.

### Distribution of compliance with spectacles according to presenting visual impairment

Spectacle compliance was highest among participants with moderate VI (19, 76%), followed by those with mild VI (11, 50%) and the least among those with no VI (9, 40.9%). Analysis revealed a statistically significant difference between spectacle compliance and presenting VI (χ^2^ = 14.63, *df* = 6, *p* = 0.023) as shown in Table 3.

**TABLE 3:** Distribution of compliance with spectacles according to presenting visual impairment.

Compliance with spectacle wear	Presenting visual impairment						Total	%	*p*
	Normal		Mild		Moderate				
	*n*	%	*n*	%	*n*	%			
Wearing spectacles	9_a_	40.9	11_a, b_	50.0	19_b_	76.0	39	56.5	-
Not wearing but brought them to school	1_a_	4.5	5_a_	22.7	2_a_	8.0	8	11.6	-
Left them at home	6_a_	27.3	5_a_	22.7	1_a_	4.0	12	17.4	-
Misplaced them	6_a_	27.3	1_a_	4.5	3_a_	12.0	10	14.5	-

**Total**	**22**	**100**	**22**	**100**	**25**	**100**	**69**	**100.0**	**0.023**

Note: Significant at *p* < 0.05.

Each subscript letter denotes a subset of VI before correction categories whose column proportions do not differ significantly from each other at the 0.05 level. a, did not differ significantly among visual impairment groups; b, did not differ significantly among visual impairment groups.

### Distribution of compliance with spectacles according to age group

There was no statistically significant association between spectacle compliance and age group (χ^2^ = 1.77, *df* = 3, *p* = 0.621); despite the 18–35 years olds having slightly better compliance levels than the 0–17 years olds, as shown in Table 4.

**TABLE 4:** Distribution of compliance with spectacles according to age group.

Compliance with spectacle wear	Age group				Total	%	*p*
	Children		Youth				
	*n*	%	*n*	%			
Wearing spectacles	21	55.3	18	58.1	39	56.5	-
Not wearing but brought them to school	3	7.9	5	16.1	8	11.6	-
Left them at home	8	21.1	4	12.9	12	17.4	-
Misplaced them	6	15.8	4	12.9	10	14.5	-

**Total**	**38**	**100**	**31**	**100**	**69**	**100.0**	**0.621**

Note: Significant at *p* < 0.05.

## Discussion

In this study, compliance with spectacle wear among learners with hearing impairment was assessed 3 months after the provision of spectacles. This study found high compliance with spectacle wear among the participants, with the majority having moderate VI before dispensing spectacles. The findings suggest that learners with visual impairments in schools for the deaf have a higher need for visual aids because they rely on optimal vision to function. Visual impairments may affect their academic performance, and providing them with spectacles significantly improves their vision, which can lead to better academic outcomes and quality of life (Kwarteng et al. 2024; Wu, Feng & Zhang 2023). Schools need to prioritise vision screening and the provision of spectacles for learners with hearing impairment not only to improve vision but also to enhance their educational and learning opportunities. While the primary aim is to address vision issues, the improved vision can positively impact their access to educational materials, ensuring parity with their peers without visual impairment. This is vital as learners with hearing impairment heavily rely on visual cues for daily activities, and better vision can significantly benefit their learning experiences (Kwarteng et al. 2022c; Pehere et al. 2019).

The level of compliance (56.5%) with spectacle wear in this study was higher compared with the overall compliance (40.14%) in a review study of learners in mainstream schools (Dhirar et al. 2020). This indicates that learners in the current study may have a higher level of motivation or perceived benefit from wearing spectacles compared to learners in mainstream schools because of their reliance on good visual cues for better communication (Pehere et al. 2019). In Ghana, a similar study among learners in a mainstream school found that 33.7% of learners wore their spectacles during the time of assessment (6 months) (Ilechie et al. 2019). It is possible that the relatively shorter reassessment time for compliance in the current study (3 months) could be the reason for the higher compliance compared to the Ghanaian study. Furthermore, the participants in the Ghanaian study used a prescription determined by self-refraction, whereas the learners in the current study received ready-made or custom-made spectacles determined by the optometrist. It is possible that the prescription determined by self-refraction could have been less accurate than the spectacles prescribed by the optometrist, thereby resulting in better compliance in the current study.

Higher compliance rates of 71.6% and 73.7% have been reported in studies with reassessment times of 1 year and 10 months, respectively (Khandekar, Gogri & Al Harby 2002; Yabumoto et al. 2009). The compliance rates reported in previous studies with longer reassessment times suggest that factors other than reassessment time play a vital role in compliance with spectacle wear. These factors include the education provided to the learners and their teachers before the provision of spectacles. Therefore, it is important to consider the type of spectacles used and the education provided when assessing compliance rates.

It is worth noting that adherence to spectacle use varies across different African countries, with some recording higher compliance rates than others. Studies conducted among mainstream school learners in Botswana, Nigeria and Malawi reported spectacle compliance of 60.1%, 59% and 69%, respectively, which is higher than what is recorded in this current study (Brien Holden Vision Institute 2019; McCormick et al. 2019). The major reasons reported for the high compliance were the short (3 months) duration for reassessment, the urban location of study centres and varying frame designs (Brien Holden Vision Institute 2019; McCormick et al. 2019). In other developing countries such as Oman (Khandekar et al. 2002), a high (71.6%) spectacle compliance rate has been reported. In contrast, other studies in Africa have reported lower (< 40%) spectacle compliance levels (Congdon et al. 2008; Wedner et al. 2008). This variation in spectacle compliance rates could be attributed to factors such as access to eye care services, cultural beliefs and socioeconomic status (Morjaria, McCormick & Gilbert 2019).

Presenting VI of learners influenced the varying compliance levels across studies (Morjaria et al. 2019; Pavithra et al. 2014), where learners with poorer uncorrected vision displayed better spectacle compliance. Therefore, it is crucial to consider the presenting VI when comparing with similar studies, as it can affect compliance rates and ultimately impact the study’s findings. Furthermore, the types of spectacles, such as ready made and custom made, used in the studies may have contributed to the variations in compliance levels. Although both types of spectacles have proven to provide good visual outcomes (Asare & Morjaria 2022; Morjaria et al. 2017; Pearce 2014), their compliance levels have also been generally low among school learners (Pearce 2014). Comparison between the compliance level among the two types of spectacles could not be ascertained by this study because of the significant difference in the number of dispensed custom-made spectacles compared to pop-in spectacles.

There were more females than males at the time of the distribution of URE. Also, females had a high level of compliance compared to their counterparts although the differences were not statistically significant. Males, on the other hand, had a higher rate of misplacing spectacles than females; this finding was also not statistically significant. These findings suggest that sex differences do not play a role in the use and care of spectacles, and interventions should not be targeted at a particular sex. The study highlights the importance of educating both males and females on the proper use and care of spectacles, as well as the need for interventions that are not gender specific. This finding is similar to a study by Ilechie et al. (2019), which reported no statistically significant difference between the sexes on compliance with spectacle wear among learners in mainstream schools in Ghana. Several studies (Barria von-Bischhoffshausen et al. 2014; Holguin et al. 2006; Khandekar et al. 2013; Manny et al. 2012; Morjaria et al. 2019; Pavithra et al. 2014; Wedner et al. 2008; Yabumoto et al. 2009) have also reported similar findings among learners in mainstream schools across the globe.

In contrast, several studies among mainstream schoolchildren have shown that there is a statistically significant difference between compliance levels among females and males, with females being more compliant with spectacles wear (Aldebasi 2013; Congdon et al. 2008; Gogate et al. 2013; McCormick et al. 2019; Morjaria et al. 2019). The variation in findings between studies could be because of cultural and societal factors that influence attitudes towards wearing spectacles among male and female learners in different settings (Morjaria et al. 2019). The difference in compliance levels between males and females may also be influenced by the design and fit of the spectacles, as well as the type of visual impairment being corrected. The relationship between compliance with spectacle wear and sex is still underexplored in Africa and may vary depending on the population studied. It is clear, though not statistically significant, that females are more compliant than males. It would be interesting in a follow-up study to explore the factors that influence this observation.

There was no statistically significant difference in compliance with spectacle wear among young learners and old learners. This suggests that both age groups are capable of complying with spectacle wear even though there may be other factors that influence their behaviour. This finding is supported by Morjaria et al. (2019) who reported no consistency in compliance with spectacle wear among age groups in similar studies. Varying results have been reported with some studies (Khandekar et al. 2002; Manny et al. 2012) finding high spectacle compliance among older learners while other studies (Barria von-Bischhoffshausen et al. 2014; Holguin et al. 2006; Pavithra et al. 2014) have found better compliance among younger learners.

These inconsistencies in exploring the link between various demographic variables and compliance highlight the need for further research into these factors to improve spectacle compliance among school learners in Africa. The influence of other factors such as the type of spectacle, duration of reassessment and UCVA, among others also warrant further investigation. Understanding these factors can help educators and health practitioners develop effective strategies and design interventions to improve compliance rates, ensuring that learners receive the vision correction they need to succeed academically and personally.

### Limitations

The compliance assessment period was shorter compared to other studies. The Ghana Education Service’s use of the 2022 academic calendar had an impact on this study’s timetable because the data collection was carried out during the COVID-19 pandemic and promotional term. However, because of the prospective nature of the study, follow-up on the learners’ compliance with spectacle wear among those who had not completed school is possible. Despite the short compliance assessment period, the study results provide valuable insights into compliance with spectacle wear among learners with hearing impairment. However, the impact of external factors, such as the academic calendar, should be considered in future studies to ensure a more comprehensive comparison to similar studies.

## Conclusion

Compliance with spectacle wear was high among learners with hearing impairment in this study compared to a previous study in Ghana and other parts of the continent. The level of compliance shown by learners was found to be influenced by presenting VI; however, neither sex nor age group had a significant influence on spectacle wear compliance. The long-term effects of improved compliance with spectacle wear on academic performance and overall quality of life remain for future investigations.
